# Soil as a Battlefield and a Reservoir: Linking Soil Components to the Epidemiology of Soilborne Plant Diseases

**DOI:** 10.1007/s00248-026-02810-6

**Published:** 2026-06-13

**Authors:** David Pires, Florabelle Castañeda, Leny Galvez, Mark Angelo Balendres

**Affiliations:** 1 Mediterranean Institute for Agriculture, Environment and Development (MED) & Global Change and Sustainability Institute (CHANGE), Institute for Advanced Studies and Research, University of Évora, Évora, Portugal; 2 National Institute for Agricultural and Veterinary Research (INIAV, I.P.), Oeiras, Portugal; 3 Department of Biology, College of Science, De La Salle University, Manila, Philippines; 4 Plant and Soil Health Research Unit, Center for Natural Science and Environmental Research, De La Salle University, Manila, Philippines; 5 Department of Agriculture, Philippine Fiber Development Authority, Quezon City, Philippines; 6 Tasmanian Institute of Agriculture, University of Tasmania, Hobart, Australia

**Keywords:** Biological control, Microbial ecology, Plant disease triangle, Soil organic matter, Soil suppressiveness

## Abstract

This paper focuses on how microbial diversity, soil organic matter, and soil structure influence the activities of soilborne pathogens and plant disease epidemiology. Microbial diversity, soil organic matter, and soil structure are soil components that can reshape plant–pathogen–soil interactions by altering nutrient dynamics and the composition of the soil microbiome. When beneficial microorganisms are enriched in soil ecosystems, suppression of soilborne pathogens may be enhanced, thereby decreasing disease incidence and severity. However, microbial diversity, soil organic matter, and soil structure may also promote pathogen growth or facilitate cooperative microbial interactions that improve pathogen persistence, thereby elevating disease risk. Future progress requires a shift from descriptive surveys toward functional and predictive approaches, as these soil components influence epidemiological processes that can either suppress or intensify the development of plant diseases caused by soilborne plant pathogens. Rather than acting as deterministic drivers of disease outcomes, microbial diversity, soil organic matter, and soil structure modify the ecological context in which host–pathogen interactions occur, altering the likelihood of pathogen establishment, persistence, and transmission. This paper highlights the importance of soil management in regulating microbial community dynamics and supporting plant disease control within this probabilistic ecological framework.

## Introduction

Soil is a densely populated and highly competitive ecosystem in which pathogen establishment depends on successful invasion into an existing microbial community. Classical plant disease epidemiology emphasized the disease triangle (host, pathogen, environment), often reducing soil to a passive physical matrix for root growth [1]. Advances in microbial ecology now demonstrate that microbial diversity is a critical biological factor that modulates all three components of the plant disease triangle principle, influencing pathogen survival, host susceptibility, and environmental filtering [2]. Importantly, these interactions do not imply deterministic relationships between microbial diversity and disease outcomes. Instead, soilborne disease emergence reflects the outcome of complex ecological processes, including community assembly, environmental filtering, resource competition, and trophic regulation within the soil food web [3, 4]. From this perspective, soil suppressiveness can be viewed as an emergent property of ecological interactions occurring across multiple spatial and temporal scales. In this review, the metaphor of soil as both a “reservoir” and a “battlefield” is used as a conceptual heuristic to illustrate conducive and suppressive soils, respectively, and how microbial communities may either facilitate or constrain pathogen establishment. However, this framework is not intended to imply binary soil states; rather, it highlights a continuum of ecological conditions in which microbial diversity, soil organic matter (SOM), and soil structure collectively shape pathogen dynamics and disease risk. Consistent with this view, numerous studies have shown that soil’s suppressiveness against plant diseases is linked with the diversity of soil microorganisms, which act against pathogens in several ways, including antibiosis, parasitism, competition, and induced resistance [5–8].

Beyond microbial diversity, SOM plays a central role in structuring belowground biological activity. As a primary energy source for the soil microbiome, SOM fuels microbial growth and interaction [9]. Through microbial decomposition, SOM supports plant growth and defense by releasing nutrients, particularly nitrogen and phosphorus, in forms that plants can readily absorb [10]. As a result, aside from the naturally occurring organic matter in the soil, supplemental organic fertilizers are used to enhance soil health and increase the soil’s suppressiveness to plant diseases.

Another important factor that affects microbial communities and their interactions with SOM is the arrangement of mineral particles, organic matter, and pore spaces into aggregates of various sizes, which dictates soil structure. Soil texture and soil structure affect how water, air, and microbes move. But soil structure changes more easily with farming practices and biological activity, so it plays a bigger role in how plant diseases develop and spread. Porosity and variations in pore sizes profoundly influence soil microbial ecology by creating variations of microhabitats with differing nutrient requirements. Fine pores or micropores tend to remain water-filled and anaerobic, while large pores or macropores drain and aerate quickly; intermediate pores provide a good balance of oxygen, moisture, and carbon for microbial communities [11, 12]. By shaping microbial community structure, soil pore characteristics ultimately influence plant–microbe interactions. While commonly recognized factors such as soil pH have been shown to exert a significant influence on soilborne disease incidence and severity [13, 14], this review specifically focuses on microbial diversity, organic matter, and soil structure. These three components were selected because they are highly dynamic, deeply interconnected, and directly responsive to agronomic management, making them key levers for engineering soil suppressiveness.

This paper reviews the current landscape of primary research on how microbial diversity, SOM, and soil structure influence the activities of soilborne pathogens and plant disease epidemiology. This paper also discusses strategies to improve soil management for controlling soilborne plant diseases and outlines future research directions. Future progress will require a shift from descriptive surveys, which typically catalog microbial taxonomy and correlate community composition with disease incidence, toward functional and predictive approaches. While descriptive studies have established the foundational link between soil microbiomes and plant health, they often cannot distinguish between correlation and causation [15, 16]. A shift is needed to mechanistically understand how specific microbial traits and metabolic functions actively suppress pathogens. By integrating these functional insights with epidemiological modeling, predictive approaches aim to forecast disease risks, identify robust indicators of soil suppressiveness, and guide precision disease management strategies.

## Influence of Soil Microbial Diversity and Function on Disease Suppression

High microbial diversity is frequently associated with reduced pathogen invasion in observational studies, a pattern theoretically supported by mechanisms described by the insurance hypothesis, niche saturation, and community assembly theory [17–19]. The insurance hypothesis proposes that higher biodiversity increases the probability that some community members can maintain ecosystem functioning under disturbance, whereas niche saturation suggests that diverse communities occupy a greater proportion of ecological niche space, leaving fewer resources available for invading pathogens [20, 21]. Within these frameworks, diverse communities are thought to occupy a larger portion of ecological niche space, limiting resource availability and opportunities for invading pathogens to establish [22].

Nevertheless, taxonomic diversity alone does not determine functional outcomes in a deterministic manner; rather, disease suppression emerges from the interaction between community composition, functional traits, and environmental context. This distinction reflects the difference between taxonomic diversity and functional diversity in microbial communities. While microbial diversity is broadly defined as the composition, relative abundance, and functional differentiation of all microbial communities within an environment, much of the current literature, and consequently the focus of this section, primarily concerns variation in bacterial community composition due to historical methodological constraints [23, 24]. In many soils, functional redundancy allows multiple taxa to perform similar ecological roles, meaning that high species richness does not necessarily translate into stronger pathogen suppression. Viewed through the lens of community assembly theory, a highly diverse soil may possess extensive functional redundancy that stabilizes the ecosystem against abiotic disturbances, yet lack the specific antagonistic traits required to filter out a particular invading pathogen [25–27].

Thus, the successful establishment of a pathogen is dictated not just by niche saturation, but by deterministic environmental filtering imposed by the resident functional guilds [28]. Conversely, the presence of specific functional groups or keystone taxa capable of producing antimicrobial compounds, parasitizing pathogens, or inducing plant defenses may disproportionately influence disease outcomes [29]. While the presence of specific functional groups capable of producing antimicrobial compounds is critical, recent phenogenomic insights into the *Trichoderma* genus demonstrate how evolution has sculpted core genomic traits for mycoparasitism and metabolic versatility [30]. This supports the view that functional traits, rather than species richness per se, dictate the success of microbial allies in the soil “battlefield”.

While broad diversity–disease relationships are often correlative, this protective function is reinforced by experimentally validated findings that greater probiotic diversity can enhance pathogen suppression; for instance, diverse *Pseudomonas* consortia in the tomato rhizosphere were found to remain viable and reduce *Ralstonia solanacearum* densities more effectively than less diverse communities, mechanistically driven by intensified resource competition and interference [31]. This has been further corroborated by recent synthetic community (SynCom) experiments, where researchers constructing 21 distinct SynComs across a diversity gradient demonstrated that high bacterial diversity directly reduced the incidence of *Fusarium* root rot by outcompeting the pathogen and forming physical barriers on the root surface [32]. Similarly, recent experiments utilizing minimal cross-kingdom SynComs have demonstrated that microbial consortia can actively remodel the rhizosphere, enriching beneficial taxa like *Mortierella* to successfully suppress wheat crown rot under pathogen pressure [33]. These processes can be interpreted through the lens of environmental filtering and priority effects, where established microbial communities modify local conditions and resource availability in ways that influence the success of later-arriving organisms, including pathogens [34, 35].

However, this community-level suppression is non-specific and can paradoxically constrain disease management efforts. For example, Nishisaka et al. [36] demonstrated that the wheat fungal pathogen *Bipolaris sorokiniana* was strongly suppressed by the biocontrol strain *Pseudomonas inefficax* CMAA1741 in low-diversity soils. In high-diversity soils from the same wheat field, however, the same inoculant failed to significantly reduce disease, as measured by disease severity index and plant growth metrics, indicating that high background microbial diversity can hinder the establishment and efficacy of introduced inoculants. This pattern is further supported by a study showing that high native soil microbial diversity can restrict the survival and impact of introduced bacterial inoculants, such as *Bacillus* spp., via resource competition and niche occupation, particularly in less-disturbed soils [37].

From an epidemiological perspective, these processes contribute to both general and specific disease suppression, which interact to dictate distinct stages of a pathogen’s life cycle. General suppression is largely driven by total microbial biomass and widespread resource competition, acting as a broad environmental filter that primarily limits initial inoculum buildup and pathogen persistence [5, 38]. Because general suppression relies on the collective competitive pressure of the whole community, it is generally non-transferable between soils. In contrast, specific suppression is mediated by defined antagonistic taxa or narrow functional guilds (e.g., antibiotic producers or obligate hyperparasites) [39]. These targeted mechanisms more directly reduce infection probability and transmission efficiency by actively attacking the pathogen during rhizosphere colonization [40]. Both modes of suppression must operate synergistically to raise the threshold required for an epidemic [41].

In field and greenhouse experiments tracking banana *Fusarium* wilt, disease suppressiveness was directly linked to the enrichment of root-associated *Pseudomonas* consortia and genotype-specific microbiomes that actively manipulated the soil microbiome and suppressed pathogen survival [42, 43]. Extending this concept across pathosystems, recent work on *Sclerotinia sclerotiorum* further demonstrates that suppressive soils are likewise driven by specific, transferable microbiome assemblages enriched in antagonistic taxa. In canola (*Brassica napus* cv. InVigor R 4022P), Han et al. [44] showed that disease suppression was microbiome-mediated and linked to the enrichment of key bacterial and fungal biocontrol groups, particularly *Bacillus* and *Streptomyces*, whose antagonistic activity constrained pathogen growth and infection, and which were absent in Sclerotinia stem rot–conducive soils.

Within this framework, the soil food web emerges as an additional regulatory layer of the battlefield. A comprehensive view of microbial diversity must therefore extend beyond bacteria and fungi to include the higher trophic levels that regulate them. Free-living nematodes, as integral components of the soil food web, contribute to disease suppression through multiple mechanisms. Their feeding is broad and non-selective, often favoring certain microbial groups such as gram-negative bacteria [45]. By grazing on microbial populations, nematodes can restructure microbial interaction networks, alter competitive hierarchies, and accelerate nutrient turnover through the release of excreted nutrients, thereby reinforcing both competitive and antagonistic pressures within the microbiome [45–49].

**Fig. 1:**
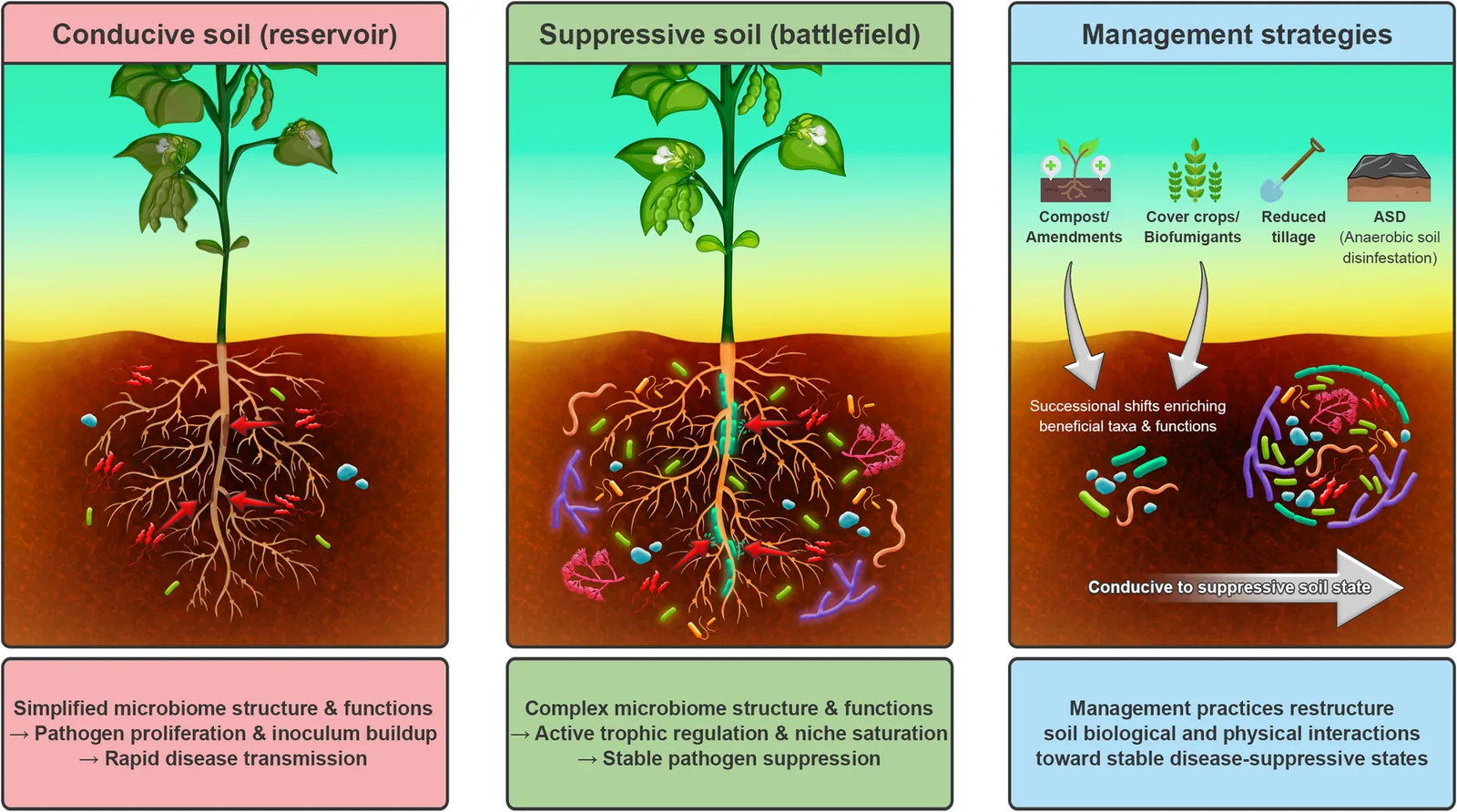
Conceptual framework illustrating how soil biological diversity and management can influence the epidemiology of soilborne plant pathogens. Rather than representing discrete soil states, the “reservoir” and “battlefield” concepts illustrate contrasting ends of a continuum of ecological conditions. Left panel: soils with limited microbial competition or trophic regulation may allow pathogen populations to accumulate (increased inoculum buildup and infection probability) and function as inoculum reservoirs. Middle panel: soils characterized by complex microbial communities and active soil food web interactions may constrain pathogen establishment and transmission. Right panel: sustainable agronomic practices can shift soil ecological conditions by restructuring microbial communities and trophic interactions, thereby modifying epidemiological parameters such as pathogen reproduction potential and infection probability

Similarly, soil protists act as key microbial predators within the soil food web, selectively feeding on specific taxa of bacteria and fungi to modulate community composition and plant–microbiome interactions, thereby influencing microbiome‑mediated disease suppression [50, 51]. Together, these mechanisms reduce the accumulation of soilborne pathogens, increasing the threshold required for disease outbreak (Fig. 1).

Despite strong conceptual support, translating microbial diversity into predictive epidemiological frameworks that forecast soilborne disease risk remains challenging. A central difficulty lies in disentangling causation from correlation in observational datasets: soils with high microbial diversity often exhibit lower disease incidence, but it is unclear whether diversity mechanistically drives pathogen suppression through microbial functional traits (e.g., antagonism, nutrient cycling) or whether healthy plants, through carbon-rich root exudation, promote a complexified rhizosphere community via microbial recruitment [36, 52]. Moreover, disease suppression does not scale linearly with diversity. Evidence increasingly supports a “diversity–function saturation” effect, in which additional diversity beyond a threshold yields diminishing returns for pathogen suppression, complicating the definition of actionable diversity targets for management [5, 10].

Methodological constraints further limit inference. Taxonomic sequencing provides information on community composition but offers limited insight into functional capacity. Limitations inherent to gene sequencing approaches in microbial ecology further constrain the reliability of functional inference. These constraints are particularly evident in metagenomic analyses. For instance, metagenomics often permits only indirect prediction of fungal disease occurrence through bacterial community signatures, whereas direct functional annotation of fungi remains limited [53]. While previous generation gene chip technology could annotate some fungal functions, this technology is outdated and has largely been abandoned [54, 55]. For example, two soils with similar diversity metrics may differ substantially in their ability to suppress disease-causing members within genera such as *Rhizoctonia* or *Fusarium*, depending on functional traits rather than species richness per se [56]. Finally, soil heterogeneity obscures key interactions: bulk soil measurements frequently fail to capture the fine-scale dynamics occurring in the rhizosphere, where pathogen infection, microbial antagonism, and host defense intersect. This challenge is typically addressed in studies by targeting rhizosphere soil, which allows researchers to better resolve the microbial and functional interactions that underpin disease suppression.

Soil microbial diversity, therefore, represents more than a reservoir of taxa; it reflects the ecological organization of microbial communities whose collective functions influence pathogen dynamics. While high diversity is frequently correlated with reduced disease incidence, outcomes ultimately depend on community composition, functional traits, trophic interactions, and environmental context [57, 58]. In this sense, the concept that diverse soils may act as biological buffers or epidemiological filters that dampen pathogen invasion should be viewed as a theoretical extrapolation rather than an absolute rule. The “battlefield” metaphor used throughout this review therefore represents a conceptual illustration of intensified ecological interactions rather than a discrete or deterministic soil state, emphasizing that suppressiveness arises from dynamic and context-dependent community processes [5, 8, 56].

Overall, the ability of soil microbial communities to suppress pathogens depends not only on diversity per se but also on functional traits, trophic interactions, and environmental context. High microbial diversity enhances general and specific suppression, but outcomes are codetermined by the composition, activity, and ecological interactions of resident microbiota under prevailing soil conditions.

## Influence of Soil Organic Matter on Disease Suppressiveness

SOM can enhance soil suppressiveness by limiting the growth and activity of phytopathogens through increases in the diversity, abundance, and functional activity of beneficial microbial communities. For instance, animal manure and waste products, organic-based fertilizers, and green manures are examples of SOM amendments used to manage anthracnose disease in vegetables [59]. Importantly, SOM amendments influence both general and specific suppression mechanisms. By increasing the overall carrying capacity and basal respiration of the soil microbiome, labile SOM fuels general suppression, intensifying carbon competition and thereby restricting pathogen inoculum buildup in the bulk soil. Simultaneously, specific chemical constituents of SOM, or the unique microbial consortia introduced by mature composts, can trigger specific suppression. This includes the selective enrichment of highly antagonistic taxa or the induction of systemic resistance in the host plant, which lowers the infection probability at the root interface [60, 61]. Recent high-throughput bioassays of commercial composts have experimentally confirmed this specific suppression, demonstrating that compost efficacy against pathogens like *R. solani* and *Globisporangium ultimum* is driven by the enrichment of specific bacterial genera (e.g., *Algoriphagus*, *Luteimonas*, *Sphingopyxis*) rather than broad physicochemical properties or total microbial biomass alone [62]. For this reason, organic amendments are commonly applied to build SOM and strengthen disease-suppressive soil conditions. However, Bonanomi et al. [63] reported variable outcomes across studies: 45% observed improved soil suppressiveness, 35% found no significant effects, and 20% documented increased disease incidence. The suppressive potential of an organic amendment depends on both its quality and its interaction with the existing soil microbiome. In general, more readily decomposable materials are effective in stimulating microbial activity [9]. Microbial genera (e.g., *Microvirga*, *Acinetobacter*, *Streptomyces*, *Bradyrhizobium*, and *Bacillus*) have been linked to plant growth promotion, whereas *Ureibacillus*, *Thermogutta*, and *Sphingopyxis* have been associated with suppressive composts [64].

A meta-analysis by Silva and Canellas [65] further indicated that increasing organic matter through sustainable biological approaches can reduce pest and disease pressure. Vermicompost, for example, supplies essential macro- and micronutrients (e.g., Ca, Mg, Zn, B, P, K, and N), along with beneficial microorganisms such as nitrogen-fixing and phosphate-solubilizing bacteria [66]. It also contains plant growth-regulating compounds, including indole-3-acetic acid, gibberellic acid, and kinetin [67]. Similarly, the microbial community in composted tea can suppress soilborne pathogens through competition for resources, parasitism, antimicrobial metabolite production, and the induction of systemic resistance in plants.

In addition, humic substances (HS) may indirectly strengthen plant defenses by limiting pathogens through inhibitory and antagonistic effects. The HS have been shown to support host resistance by increasing secondary metabolite production and eliciting plant defense responses. They may also influence secondary metabolite production, thereby eliciting plant defense responses and improving resistance [68]. HS were reported to be highly effective (75.9% reduction compared to control) in pest and disease control, although performance depends strongly on their composition and the applied concentration [63]. Overall, organic matter sources vary in their effectiveness against pests and diseases due to multiple contributing factors, including material origin, molecular composition, the target organism, and the crop system involved.

In contrast to reports that SOM enrichment promotes beneficial microbial communities, Du et al. [69] demonstrated that increasing agricultural SOM can also elevate the proportion of fungal phytopathogens, as determined through fungal internal transcribed spacer (ITS) amplicon sequencing. Their six-year fertilization experiment assessed the effects of repeated organic amendments (crop straw and fresh manure) on soilborne fungal pathogen populations. The authors reported that crop straw and cattle manure amendments, both high in soil organic carbon (SOC), increased the relative abundance of the phytopathogens *Monographella* and *Magnaporthe* in grain-producing areas. Pig manure amendment increased the relative abundance of phytopathogenic genera *Penicillium*, *Devriesia*, and *Pestalotiopsis* in wheat–maize rotation plots [69, 70]. Organic material applications may introduce a greater diversity and abundance of potential pathogens [71, 72], which can compete with beneficial microorganisms. Because organic amendments can also create favorable conditions for pathogen growth, proliferation, and colonization, they may ultimately contribute to the development of soilborne diseases [73].

**Fig. 2:**
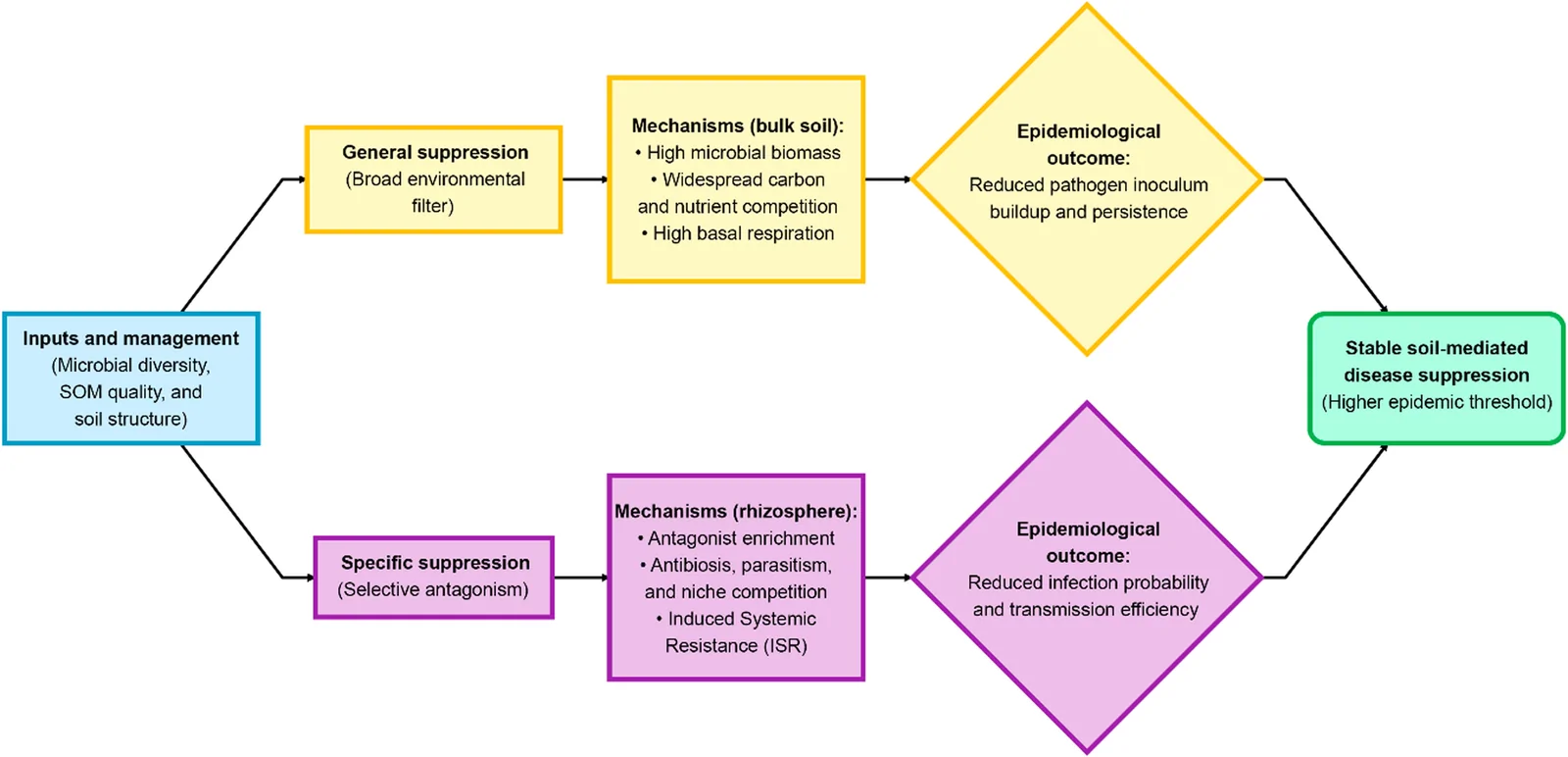
Diagram linking soil suppression mechanisms to pathogen epidemiology. Soil inputs and management (microbial diversity, SOM quality, and soil structure) drive two complementary pathways of pathogen regulation. General suppression operates in bulk soil as a broad environmental filter, primarily limiting inoculum buildup and pathogen persistence via high microbial biomass, widespread resource competition, and basal respiration. Specific suppression acts in the rhizosphere through targeted processes, including antagonist enrichment, antibiosis, parasitism, niche competition, and induced systemic resistance (ISR), to reduce infection probability and transmission efficiency. Together, these complementary pathways raise the epidemiological threshold, promoting stable plant disease suppression

Furthermore, evidence suggests that high SOC conditions may promote more positive microbial interactions, such as cooperation and facilitation, rather than competitive relationships among phytopathogens [74]. These interactions are thought to arise through several mechanisms, including, but not limited to, increased SOC, which enhances the availability of diverse carbon substrates, thereby reducing direct resource competition and enabling metabolic complementarity, and greater microbial biomass and diversity. Wei et al. [75] further showed that differences in the initial soil microbiome can influence disease outcomes. Collectively, these findings highlight the importance of managing fertilization and organic amendment strategies to regulate microbial community dynamics and support plant disease control. This multi-layered defense system can be conceptualized by linking specific microbial and chemical drivers to key stages of pathogen epidemiology (Fig. 2).

SOM and its management mediate disease suppression by modulating microbial community composition, metabolic activity, and interactions, while also influencing soil physicochemical properties such as nutrient availability and moisture. The suppressive effect of SOM is therefore a product of tightly linked microbial and abiotic factors.

## Influence of Soil Structure on Pathogen Activity

Fungal pathogens uniquely explore the soil matrix via hyphal extension, allowing movement through both water-filled and air-filled pores, unlike many bacteria, which rely on continuous water films [76]. However, hyphal growth is constrained by soil physical architecture, with pore size, connectivity, and tortuosity governing the rate and geometry of spread [77]. Experimental monitoring shows that fungal spread is strictly dictated by the microarchitecture of the pore space. Interestingly, the dispersal and environmental adaptation of beneficial fungi are further enhanced by diverse reproductive strategies; for example, *Trichoderma* species have evolved to disperse via water droplets and air, allowing them to navigate heterogeneous environments more effectively than pathogens limited to specific moisture films or pore connectivities [30]. *Rhizoctonia solani*, a widely studied soilborne pathogen causing root rot and damping-off in numerous crops, has served as a model for linking soil structure to fungal epidemiology. Studies show that fungal spread is primarily driven by microscale pore architecture rather than bulk soil properties: well-connected air-filled pores, cracks, and biopores facilitate hyphal expansion, whereas small, tortuous pores restrict growth even at comparable biomass levels [78]. Experimental microfluidic approaches have directly visualized this phenomenon, utilizing transparent soil micromodels and live-cell imaging to demonstrate that *R. solani* hyphal spread, sub-apical branching, and thigmotropism are strictly dictated by the physical confinement and microarchitecture of the pore space [79, 80]. These findings underscore soil microstructure as a key determinant of pathogen spread, highlighting how disease risk is sensitive to management practices that alter pore connectivity. From a broader ecological perspective, the soil physical matrix dictates metacommunity dynamics by strictly regulating microbial dispersal and spatial structuring [81]. Microscale habitat heterogeneity, driven by varying pore sizes, localized moisture gradients, and discontinuous nutrient patches, creates physical refugia that can decouple predator-prey interactions within the soil food web or limit competitive exclusion [82]. Consequently, poor pore connectivity can act as an environmental filter that allows pathogens to persist in isolated micro-niches, effectively shielding them from the antagonistic pressures of a highly diverse bulk soil microbiome.

Oomycete pathogens, such as *Phytophthora* and *Pythium* species, disperse via motile biflagellate zoospores that swim through water-filled soil pores toward host roots, making soil moisture and pore connectivity key determinants of infection [83]. Zoospore movement is constrained when pore throats are smaller than their ~ 6–10 μm diameter, causing physical straining, premature encystment, and restricting dispersal to saturated soil layers or continuous macropore networks [84, 85]. Consequently, oomycete epidemiology is largely governed by soil pore architecture and moisture dynamics rather than pathogen abundance alone, highlighting soil moisture regulation and structural management as effective strategies for disease suppression.

Bacterial soil pathogens such as *R. solanacearum* disperse primarily via water films coating soil particles, with movement governed by film continuity and pore connectivity. Motility enables active navigation of heterogeneous pore networks, facilitating access to isolated microhabitats, whereas fragmented water films in drier soils restrict dispersal and shape microbial diversity and community composition [86, 87]. In addition, bacterial biofilms modify soil microstructure by coating surfaces and clogging pore throats, altering hydraulic properties, generating localized anaerobic conditions, and enhancing root attachment [88]. Together, these processes underscore the central role of soil microstructure and hydrology in regulating bacterial pathogen spread and disease risk, beyond pathogen abundance or soil chemistry alone [89, 90].

Moreover, soil aggregate size strongly shapes nematode communities. In tea plantations in China, larger soil aggregates (> 2 mm) supported higher nematode abundance, diversity, and functional activity compared to smaller aggregates, resulting in higher plant-parasitic nematode pressure and disease risk. Long-term tea cultivation reduced SOM quality and disrupted aggregation, decreasing the proportion of large aggregates and altering nematode functional groups, thereby reducing soil food web structure and indirectly favoring plant-parasitic nematodes [91]. The study underscores that managing soil aggregation and maintaining a heterogeneous pore network are critical for sustaining nematode-mediated ecosystem services and mitigating soilborne plant disease.

Soil compaction from machinery, foot traffic, or grazing alters soil structure by reducing macropores and increasing bulk density, creating stress on plants while often favoring pathogens. Compacted soils limit aeration, restrict root penetration, and trap ethylene, inducing adaptive responses such as radial swelling, reduced root hair development, and decreased cytoplasmic streaming. These changes weaken plant defenses, modify root architecture, and increase susceptibility to soilborne pathogens. Epidemiologically, compaction correlates with greater root disease severity; increased bulk density has been shown to elevate *Fusarium* and other root rot incidence, likely through hypoxia and water-saturated conditions that enhance pathogen activity and stress host roots [92]. Similarly, *Rhizoctonia* root rot severity increases under compaction due to restricted root growth and reduced biomass [93]. Compaction also shifts soil microbial communities, favoring anaerobic prokaryotes and saprotrophic fungi while reducing aerobic prokaryotes and plant-associated fungi [94]. Viewed through the lens of community stability frameworks, the destruction of macropores through compaction homogenizes the soil microenvironment, collapsing the spatial refugia that sustain microbial diversity [11, 95, 96]. This structural homogenization acts as a severe environmental filter, shifting community assembly toward a less diverse, highly specialized pathobiome [97, 98]. Without the spatial structuring required to support diverse antagonistic populations, the soil microbiome loses its functional redundancy and its capacity to buffer against pathogen proliferation [94]. These findings underscore that soil physical integrity is a key determinant of plant health and disease risk, highlighting the importance of managing compaction in integrated disease management. Beyond microbial community effects, compaction also mediates disease risk through root system architecture (RSA). RSA mediates disease risk by determining root distribution relative to pathogen inoculum. In compacted soils, roots exploit macropores or cracks to reach deeper, less pathogen-dense layers, whereas shallow, highly branched roots remain in the topsoil, increasing exposure [99, 100]. RSA also shapes microbial communities, influencing pathogen suppression or facilitation through microbial recruitment along soil gradients [101, 102]. Soil compaction and aggregate-mediated pore structure regulate the distribution and movement of plant-parasitic nematodes, which, along with fungi and oomycetes, shape the intensity and spatial patterns of root diseases. These interactions highlight that soil physical integrity, root architecture, and microbial communities collectively contribute to disease risk, severity, and crop resilience, emphasizing the need to address them in effective plant disease management.

In summary, soil structure governs pathogen dispersal, root architecture, and microbial interactions, creating spatial heterogeneity that regulates disease risk. The physical matrix and pore connectivity work in concert with microbial communities and trophic networks, demonstrating that soil architecture and biology jointly shape plant pathogen dynamics.

## Soil Management for Soilborne Plant Disease Control

If soil is a battlefield, soil management represents the strategic deployment of resources to favor beneficial allies over pathogenic adversaries. Historically, soilborne disease control relied heavily on chemical fumigation. The primary concerns associated with soil fumigation involve the use of highly toxic chemicals and their adverse atmospheric effects, though their impact on microbial community diversity is also a significant issue, as they create a biological vacuum often recolonized by opportunistic pathogens [103, 104]. However, the development and application of environmentally friendly fumigants may allow soilborne diseases to be effectively managed while mitigating undesirable effects on microbial communities [105]. For example, compounds such as dimethyl disulfide and allyl isothiocyanate have been proposed as sustainable alternatives, and environmentally friendly fumigation with ethylicin has been shown to significantly reduce soil bacterial diversity while concurrently increasing fungal diversity [106]. Contemporary approaches instead aim to manipulate the soil food web, promoting suppressive microbial communities and their trophic regulators rather than eliminating pathogens outright. By contrast, anaerobic soil disinfestation (ASD) increased bacterial diversity, optimized the core microbiome, and stimulated potential disease-suppressive agents, conferring more stable suppression of invasion and improved plant health [107]. These outcomes illustrate that effective management reshapes not only microbial composition but also trophic interactions within the soil food web, stabilizing suppressive functions through coupled microbial and faunal regulation. Mechanistic work on ASD in a cucumber damping-off system demonstrates that both altered abiotic conditions and specific microbial groups underpin disease suppression [108]. In this system, ASD increased soil pH and labile carbon availability, creating an environment distinct from untreated diseased soil. These abiotic shifts were shown to be the primary drivers of bacterial community reassembly, while the initial microbiome more strongly influenced fungal community composition [108].

Principles of conservation agriculture, including minimal soil disturbance, permanent soil cover, and diversified crop rotations, form the foundation of the shift from conventional pathogen-targeted management toward strategies that enhance soil health and microbiome-mediated disease suppression. Complementary strategies such as organic amendments (e.g., composts and biochar), ASD, and biofumigant cover crops are used to restructure microbial communities and alter soil physicochemical conditions. Rather than pursuing pathogen eradication, these approaches aim to restore soil food web complexity, in which microbial competition, antagonism, functional redundancy, and trophic regulation collectively limit pathogen survival, infectivity, and transmission efficiency [109, 110].

Biologically based soil management, however, remains less predictable than chemical control. The most persistent challenge is context dependency: practices that suppress pathogens such as *Verticillium* in one field may fail in another due to subtle differences in soil chemistry, texture, climate, or baseline microbial composition. Time lag is an additional constraint. Unlike fumigation, which rapidly reduces pathogen inoculum, the development of suppressive soils is gradual and may require multiple seasons, posing economic risks during transitional periods. A persistent challenge in biologically based management is the risk that introduced microorganisms may exhibit opportunistic or detrimental behavior under certain conditions. Addressing this, Steindorff et al. [30] utilized machine learning to identify specific traits of biosafety concern, providing a science-based framework to distinguish safe, targeted bioeffectors from potentially harmful *Trichoderma* strains, thereby increasing the predictability of biological control strategies. This is particularly relevant as most beneficial microorganisms, including *Trichoderma* and various beneficial bacteria, also rely on saprotrophic nutrition, which may inadvertently favor opportunistic pathogens or detrimental saprophytes under certain environmental conditions. Reviews on compost‑based and compost‑derived disease suppression show that disease‑suppressive composts can enhance natural suppressiveness by introducing beneficial microbiota and stimulating general suppression mechanisms such as increased microbial activity, fungistasis, competition for space and nutrients, antibiotic production, and systemic resistance [6, 7]. Moreover, because soil food webs are inherently dynamic and context-dependent, increases in trophic complexity do not guarantee uniform outcomes across systems. While direct empirical evidence remains limited, there is a theoretical risk that soilborne pathogens could adapt to persistent biological suppression or exploit transient imbalances within food web interactions, either by tolerating antagonistic compounds or exploiting alternative ecological niches.

Effective soilborne disease management, therefore, requires a paradigm shift from eradication to ecological balance. Despite challenges related to predictability and transition timeframes, integrated strategies combining organic amendments, reduced tillage, and crop diversification offer the most sustainable path forward. By managing soil as a living habitat rather than a substrate, growers can recruit indigenous microbial communities as persistent, self-regenerating defenses against soilborne diseases, reducing long-term dependence on chemical inputs. Long‑term work shows that suppressiveness is largely microbial in origin, involving antibiosis, parasitism, competition, and induced resistance [5–8].

Together, conservation agriculture, organic amendments, ASD, and other ecologically based management strategies illustrate that effective disease control emerges from the integration of soil physicochemical conditions and microbiota. Manipulating both abiotic and biotic components of the soil ecosystem can enhance suppressive functions, stabilize microbial communities, and reduce reliance on chemical interventions.

## Research Outlook and Future Progress

Future advances in soilborne disease research will require a shift from observational correlations and co-occurrence patterns toward mechanistic and predictive frameworks that explicitly link microbial community structures and functions with epidemiological outcomes. Achieving this goal will require moving beyond diversity metrics alone and integrating functional trait analyses and microbial interaction networks to better predict pathogen dynamics in soil ecosystems. In this context, integrating microbial ecology with ecological theory, particularly community assembly processes, environmental filtering, and metacommunity dynamics, will be essential for translating microbiome observations into predictive epidemiological models. For example, applying metacommunity theory to soilscapes could help explain how source-sink dynamics and pathogen dispersal among discrete soil aggregates influence the long-term stability of disease-suppressive states.

Functional metagenomics and trait-based approaches, combined with network analyses, will be central to identifying the genes, functional guilds, and keystone taxa that constrain pathogen establishment within complex pathobiomes [5–10, 97, 98]. A benchmark for this transition is the use of phenogenomics, correlating extensive phenotypic libraries with genomic data, to identify “fitness genes” that ensure the stability of beneficial microbes like *Trichoderma* in complex soil ecosystems [30]. Longitudinal, high-resolution temporal studies will further be essential to capture microbial transitions that precede disease outbreaks, enabling microbiome-informed early-warning indicators for soilborne disease outbreaks [111]. Specifically, future predictive frameworks must integrate the three core factors highlighted in this review, because shifts in soil pore networks and changes in SOM composition can simultaneously reshape microbial functional diversity and pathogen transmission pathways. By synthesizing these physical, chemical, and biological data streams, researchers can develop robust models capable of anticipating soilborne disease emergence before symptoms appear in the host.

Emerging technologies now provide the means to integrate microscale root–microbe interactions with applied precision disease management. Advanced imaging tools and synthetic microbiomes offer complementary platforms to experimentally test causal mechanisms and design pathogen-suppressive microbial communities. Translating these insights into practice will require prescriptive soil management strategies, including engineered microbial consortia, crop breeding for rhizosphere competence, predictive modeling, and improved standardization of organic amendments.

As climate change continues to intensify and the development of traditional genetic resistance in plants increasingly becomes a “losing race” against rapidly evolving pathogens, microbiome-based disease management is likely to gain increasing importance. Engineered SynComs represent a promising avenue for designing stable and multifunctional microbial defenses against soilborne pathogens. Mounting evidence suggests that the efficacy of these microbial consortia can be enhanced by targeting core metabolic pathways and evolutionary adaptations that underpin selective antagonism and environmental resilience [30, 33, 112–117]. We propose that the future of resilient agriculture lies in moving beyond host-centric traits toward the targeted design of dynamic, self-regenerating SynComs. These engineered microbiomes can be tailored to maintain functional stability under environmental stress, offering a more adaptable and proactive solution than genetic resistance alone. Together, these approaches position the soil microbiome as a manipulable interface for precision phytopathology rather than a passive background to disease emergence [5, 7, 8, 118–121]. Within this perspective, soil ecosystems can be viewed along a continuum between pathogen reservoir states and biologically competitive environments where microbial interactions limit pathogen establishment and transmission.

## Data Availability

No datasets were generated or analysed during the current study.
